# Case Report: A rare case of hepatoid carcinoma of the ovary with genomic profiling and long-term follow-up: diagnostic and therapeutic perspectives

**DOI:** 10.3389/fonc.2025.1631424

**Published:** 2025-08-22

**Authors:** Yufang Zuo, Xuan Liu, Yajun Pang, Xiaowen Chen, Xiaofang Li, Jiangsheng Gao, Bingjie Liu, Sihai Liao, Ying Zhang, Chengnong Guan

**Affiliations:** ^1^ Department of Gynecological Oncology, Affiliated Hospital of Guangdong Medical University, Zhanjiang, Guangdong, China; ^2^ Department of Pathology, Affiliated Hospital of Guangdong Medical University, Zhanjiang, Guangdong, China; ^3^ Guangdong Medical University, Zhanjiang, Guangdong, China; ^4^ Department of Obstetrics and Gynecology, Affiliated Hospital of Guangdong Medical University, Zhanjiang, Guangdong, China; ^5^ Department of Oncology, The First Dongguan Affiliated Hospital of Guangdong Medical University, Dongguan, Guangdong, China

**Keywords:** hepatoid carcinoma of the ovary, diagnosis, multidisciplinary, case report, alpha-fetoprotein

## Abstract

Hepatoid carcinoma of the ovary (HCO) is a highly uncommon and aggressive neoplasm originating from the surface epithelial cells of the ovary, characterized by hepatocyte-like differentiation. To date, most information on HCO is derived from case reports, with fewer than 50 documented cases globally. In this case report, we present a detailed account of the diagnosis, treatment, and prognosis of a patient diagnosed as having bilateral HCO, which is even rarer. Targeted next-generation sequencing revealed somatic mutations in PIK3C3 and TP53, with no BRCA1/2 alterations, and a molecular profile consistent with microsatellite stability and low tumor mutational burden. We also review the current literature to situate our findings within the broader context of existing knowledge. Given the rarity of bilateral HCO, our objective is to contribute to the existing body of knowledge by providing a comprehensive description of its clinical features, molecular characteristics, and treatment strategies. This effort may enhance understanding of this rare malignancy and offer insights to improve patient outcomes in clinical practice.

## Introduction

Hepatoid carcinoma of the ovary (HCO) is an exceptionally rare and aggressive subtype of malignant ovarian tumors, first described by Ishikura and Scully in 1987 (1). Histologically and immunophenotypically, it closely resembles hepatocellular carcinoma (HCC), often presenting with markedly elevated serum alpha-fetoprotein (AFP) levels. Due to these features, HCO is frequently misdiagnosed as metastatic HCC, particularly in patients with underlying liver disease.

HCO primarily affects perimenopausal and postmenopausal women, and is typically diagnosed at an advanced stage. Common clinical features include abdominal discomfort, high serum AFP levels, and adnexal masses on imaging (2). While scattered case reports have described the clinicopathological characteristics of HCO, it’s extremely low incidence hampers a comprehensive understanding of its pathogenesis, diagnostic criteria, and therapeutic options. Differentiating primary ovarian HCO from hepatic metastases remains especially challenging.

At present, management strategies are largely extrapolated from those for epithelial ovarian carcinoma, involving cytoreductive surgery followed by platinum-based chemotherapy (3). However, due to the scarcity of reported cases and the absence of standardized treatment protocols, no consensus has been established, and the role of molecular profiling remains unexplored.

To address these gaps, the reporting of HCO cases with integrated pathological and molecular analyses, along with long-term clinical follow-up, is critical to improving our understanding of this rare tumor and informing future management.

This report presents a case of primary HCO in a 44-year-old woman, detailing the diagnostic and therapeutic process, including clinical presentation, imaging, histopathological and immunohistochemical features, molecular profiling, and treatment response. We also conducted an extended follow-up and reviewed 47 previously published cases to summarize the clinical characteristics, therapeutic strategies, and prognostic features of HCO. This case highlights the potential value of molecular profiling and aims to contribute to the refinement of diagnostic and therapeutic approaches for rare ovarian malignancies.

## Case report

### Patient history and presentation

In September 2022, a 44-year-old woman underwent surgery for lumbar disc herniation. Routine postoperative laboratory tests revealed a markedly elevated serum AFP level of 2,000 ng/mL. Given that AFP is a key tumor marker for hepatocellular carcinoma (HCC), and considering her 30-year history of chronic hepatitis B with ongoing antiviral therapy, a primary or metastatic hepatic malignancy was strongly suspected.

**Figure 1:**
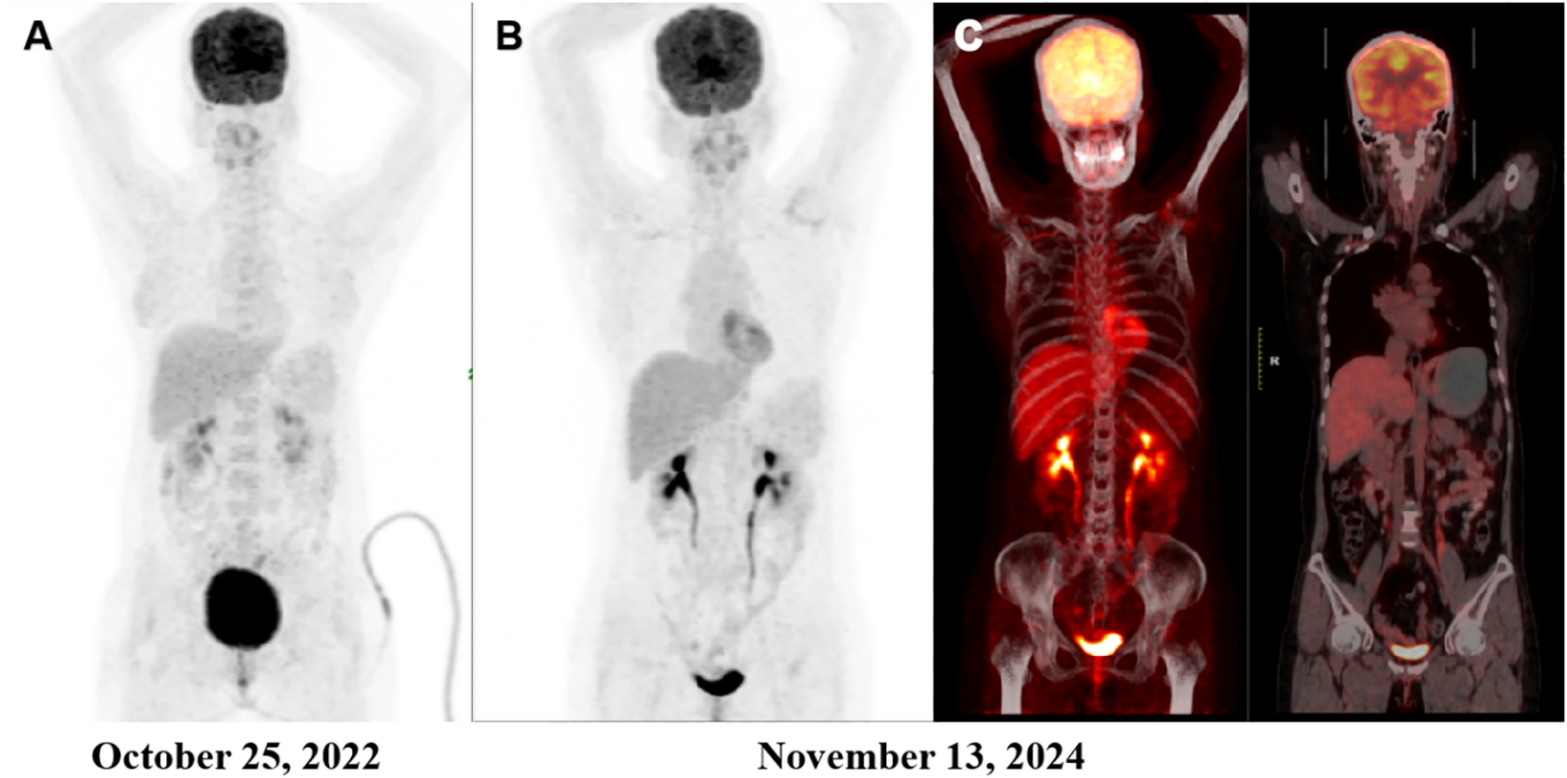
Comparison of PET/CT scans before and after treatment. **(A)** Preoperative PET/CT image showing no hypermetabolic activity in the liver. A 1.6 cm cystic lesion was noted in the right adnexal region, and a physiologically active focus was observed in the left adnexal region (SUVmax = 4.5). **(B, C)** Post-treatment PET/CT images obtained 19 months after surgery revealed no abnormal hypermetabolic activity suggestive of recurrence.

On October 21, 2022, upper abdominal MRI revealed no obvious hepatic lesions. To further evaluate potential intrahepatic or extrahepatic disease, a whole-body ^18^F-FDG PET/CT scan was performed. No abnormal metabolic activity was observed in the liver. However, a 1.6 cm cystic lesion was noted in the right adnexal region, and mildly increased FDG uptake (SUVmax=4.5) was detected in the left adnexa, consistent with physiological activity. Gynecological follow-up was recommended (**Figure 1**).

On February 28, 2023, the patient developed irregular vaginal bleeding without apparent cause. On March 20, 2023, she presented to our outpatient clinic. Her serum AFP level had risen significantly to 20,164 ng/mL. Abdominal ultrasound again showed no hepatic masses but revealed a heterogeneous mass in the right ovary measuring approximately 7.2 × 6.0 cm, suggestive of an ovarian neoplasm.

On March 30, the patient was admitted for further evaluation. Gynecological examination revealed normal external genitalia, a patent vaginal canal with moderate milky-white discharge, a smooth cervix without contact bleeding, and an anteverted uterus of normal size. A firm, moderately mobile mass approximately 7 cm in diameter was palpable in the right adnexal region, while no abnormalities were detected on the left. Transvaginal ultrasound performed the same day identified a 15 mm hypoechoic lesion in the left ovary and a predominantly solid, heterogeneous mass in the right adnexal region measuring 88 × 64 mm, raising suspicion for a malignant ovarian tumor such as a yolk sac tumor.

**Figure 2:**
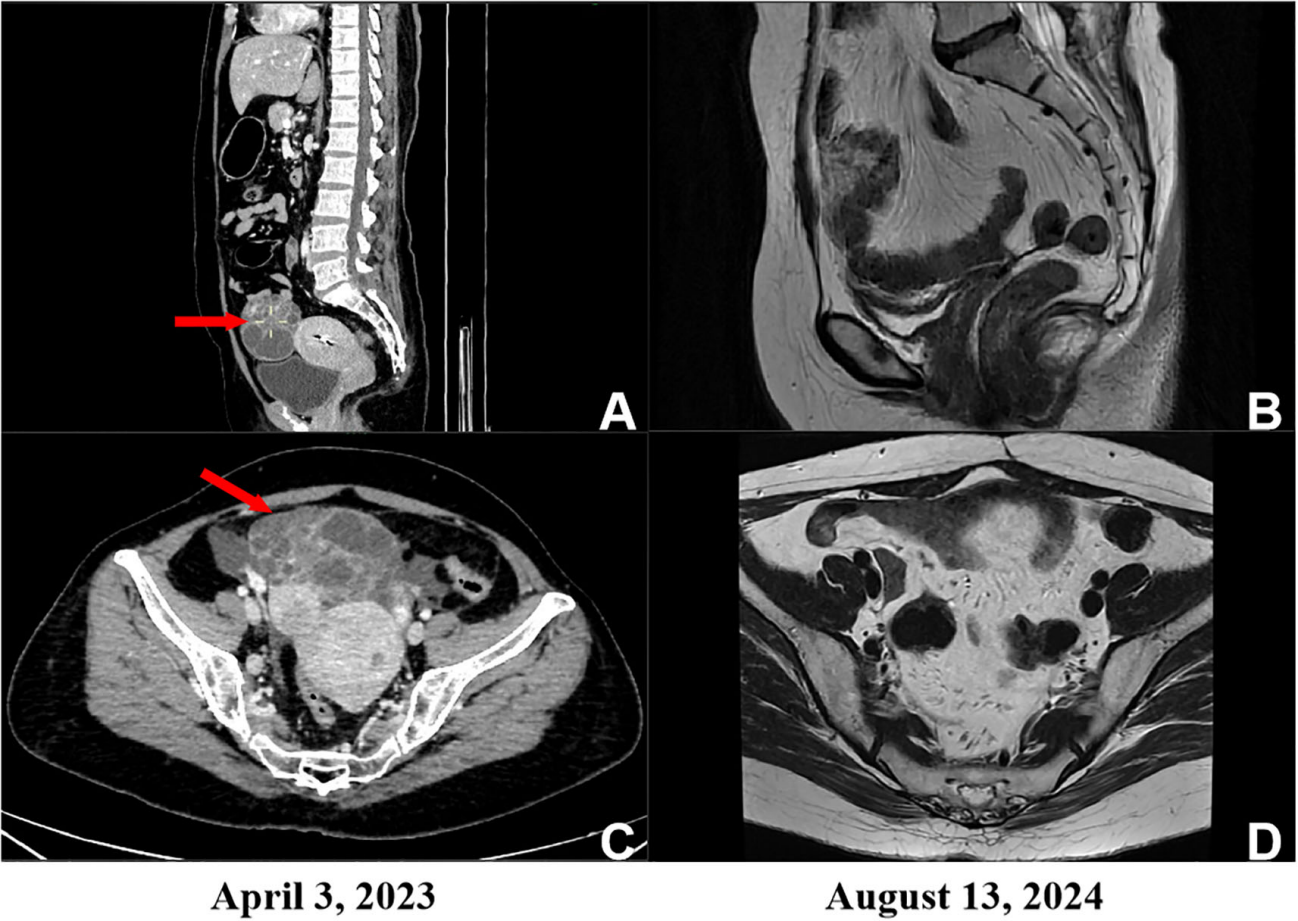
Pre- and post-operative pelvic imaging. **(A, C)** Preoperative coronal and axial CT images demonstrating a space-occupying lesion anterior to the uterus in the right pelvic cavity, highly suspicious for malignancy. **(B, D)** Corresponding coronal and axial MRI images obtained after comprehensive treatment, showing no radiologically detectable residual or recurrent disease.

On March 31, her serum AFP level had further increased to 21,130 ng/mL. Abdominopelvic CT on April 3 revealed a space-occupying lesion in the right pelvis, anterior to the uterus, highly suggestive of malignancy. Given the patient’s age, tumor morphology, and markedly elevated AFP, a right ovarian yolk sac tumor was initially considered (**Figures 2A, C**).

On April 4, 2023, after ruling out surgical contraindications, the patient underwent diagnostic laparoscopy. Intraoperative findings included a grossly normal-sized uterus. A 1.5 cm tumor was observed on the surface of the left ovary, and a 9 cm cystic-solid mass with ill-defined borders was identified in the right ovary. Scattered rice grain-like tumor implants were observed on the surfaces of the greater omentum and both diaphragms, with the largest measuring approximately 1 cm in diameter. Resection and ablation of diaphragmatic lesions were performed with assistance from hepatobiliary surgeons. No significant adhesions were noted among the omentum, intestines, and peritoneal surfaces. The liver and gastric serosa appeared smooth, without evidence of metastatic nodules.

Based on these intraoperative findings, the procedure was converted to open surgery. The patient subsequently underwent total hysterectomy, bilateral salpingo-oophorectomy, omentectomy, tumor debulking, resection and ablation of diaphragmatic lesions, placement of an intraperitoneal hyperthermic perfusion catheter, lysis of adhesions, and resection of mesenteric and left lower abdominal peritoneal implants. The operation lasted approximately 6 hours, with an estimated blood loss of 300 mL. No transfusion was required, and the procedure was completed uneventfully.

Complete macroscopic resection of all visible tumors was achieved, fulfilling the criteria for R0 resection. Based on the intraoperative findings, preoperative imaging, and postoperative immunohistochemical analysis, a final diagnosis of bilateral HCO was established. According to the 8th edition of the AJCC staging system, the tumor was staged as pT3bNxMx (**Supplementary Figure 1**).

### Immunohistochemistry results

**Figure 3:**
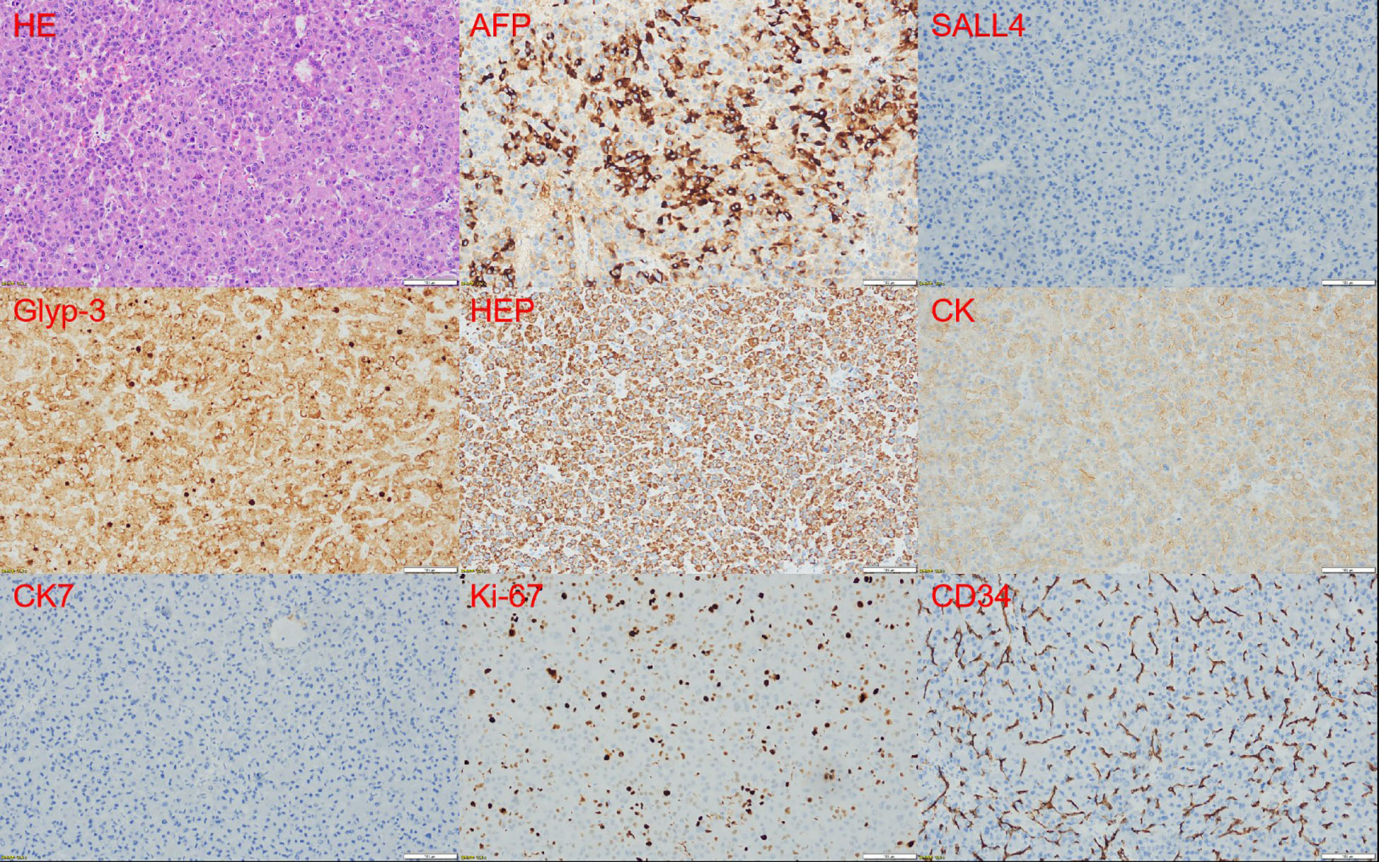
Histopathological and immunohistochemical features of the tumor. HE(200×)Staining showed eosinophilic cytoplasm with round to oval central nuclei, visible nucleoli, and distinct cell borders, similar to hepatocellular carcinoma. AFP (partial+), SALL4 (-),Glypican-3 (+),Hep (+),CK (+),CK7 (+),Ki67 (~30% in hotspot areas), CD34 (vascular +).

Immunohistochemical analysis demonstrated the following results: AFP (partially +), Glypican-3 (+), SALL4 (–), PLAP (–), OCT3/4 (–), D2-40 (–), CD30 (–), CD117 (–), Ki-67 (~30% in hotspot areas), CK (+), HCG (–), CD34 (vascular +), HepPar-1 (+), CD10 (focal +), HBsAg (–), HBcAg (–), CK7 (+), CK19 (–), Villin (+), CK20 (–), CDX2 (–), α-inhibin (luteinized cells +), Synaptophysin (–), CD56 (–). Afterward, a pathological consultation at the Cancer Center of Sun Yat-sen University confirmed the diagnosis of hepatoid carcinoma of the ovary (HCO) (**Figures 3**).

### Genetic testing results

Genomic profiling was performed on formalin-fixed tumor tissue and matched peripheral blood DNA to assess both somatic and germline alterations. Targeted next-generation sequencing (NGS) was conducted using a 437-gene cancer panel, covering approximately 1.53 Mb of genomic regions. Sequencing was carried out on a high-throughput NGS platform, and variant annotation was performed with reference to the GRCh37/hg19 human genome assembly.

Comprehensive genomic profiling identified several somatic mutations and pharmacogenetic polymorphisms in the patient. Germline mutation analysis did not reveal any pathogenic variants. Tumor-specific mutations included a promoter mutation in TERT (C.-124C>T, AF 22.07%) and missense mutations in CDKN1A (c.463C>T, p.R155C, AF 7.18%), HDAC9 (c.1335C>A, p.N445K, AF 11.41%), NCOR1 (c.2168C>A, p.P723Q, AF 2.98%), PIK3C3 (c.2183G>C, p.S728T, AF 25.81%), TNFRSF11A (c.301G>A, p.V101M, AF 25.00%), and ZNF217 (c.1333T>A, p.S445T, AF 2.02%). In addition, pharmacogenetic analysis identified a heterozygous deletion polymorphism in BCL2L11 (c.394 + 1479_394 + 4381del), a homozygous polymorphism in ERCC1 (c.354T>C, p.N118=), heterozygous polymorphisms in GSTP1 (c.313A>G, p.I105V) and NQO1 (c.559C>T, p.P187S), and a homozygous deletion polymorphism in TYMS (c.450_455delAAGTTA).

Further extended molecular testing was performed on July 10, 2023, by Geneseeq Technology Inc. (Nanjing, China). This analysis confirmed the previously identified PIK3C3 mutation and showed no pathogenic alterations in BRCA1/2. The tumor exhibited low tumor mutational burden (TMB) and was microsatellite stable (MSS).

### Treatment and follow-up

Following surgery, the patient’s serum AFP level declined to 3763 ng/ml. Routine follow-up involved periodic CT imaging and serial measurements of AFP levels. Three days after surgery, she underwent hyperthermic intraperitoneal chemotherapy (HIPEC) using normal saline as the perfusate, based on evidence supporting its benefit in selected cases of advanced ovarian cancer. Subsequently, the patient received four cycles of chemotherapy consisting of liposomal paclitaxel and carboplatin. During this period, grade 2 leukopenia was observed and managed with granulocyte colony-stimulating factor (G-CSF), with no treatment delays.

Genetic testing revealed no pathogenic mutations in the BRCA1 or BRCA2 genes, and thus PARP inhibitors were not considered appropriate at this stage. Given the tumor’s hypervascular characteristics observed intraoperatively and on imaging, and in the absence of established treatment guidelines for HCO, bevacizumab (400 mg per cycle) was incorporated into the regimen. This decision was supported by extrapolation from epithelial ovarian cancer protocols, in which bevacizumab has demonstrated clinical benefit, and further justified by the presence of a somatic PIK3C3 mutation in the patient’s tumor, which is associated with PI3K pathway activation and may contribute to angiogenesis.

The patient subsequently received three additional cycles of combination therapy with liposomal paclitaxel, carboplatin, and bevacizumab. Follow-up CT scans during this period revealed no evidence of recurrence or metastasis (**Figures 2B, D**). However, the AFP level remained above the normal range, at 42.8 ng/ml. Although clinical indications supported the continuation of targeted combination chemotherapy, the patient chose to forgo further combination chemotherapy in favor of single-agent maintenance therapy with 400 mg of bevacizumab. During targeted maintenance therapy, the patient experienced grade 1 leukopenia, which was managed with supportive treatment for leukocyte recovery.

AFP levels were monitored monthly. Although persistently above the normal range, they remained stable until the 13th month, when a gradual upward trend was noted (**Supplementary Figure 2**). To assess for recurrence, a PET/CT scan was performed on November 13, 2024, which demonstrated no hypermetabolic lesions suggestive of recurrence (**Figures 1B**). As AFP levels continued to rise, a second PET/CT scan was conducted on March 26, 2025; again, no metabolically active lesions indicative of recurrence were identified.

At the time of manuscript submission, the patient had completed 25 months of treatment and follow-up. While she remained clinically stable and exhibited no radiologically confirmed recurrence, serial AFP measurements revealed a persistent upward trend, with the most recent value reaching 889 ng/mL, raising concern for potential biochemical progression and warranting close surveillance (**Supplementary Figures 2A**). All therapeutic decisions were made with full informed consent from the patient and her family (**Supplementary Figure 3**).

## Discussion

HCO is a rare subtype of surface epithelial ovarian tumors, known for its aggressive behavior and rapid clinical progression. Despite its unique clinical features, HCO has not yet been formally recognized as a distinct pathological entity in the 2014 update of the World Health Organization (WHO) tumor classification (4). Current understanding of HCO is primarily derived from individual case reports. A review of the literature (see **Table 1**) (1–3, 5–40) indicates that 47 cases of HCO have been documented globally since it was first reported by Ishikura and Scully in 1987. HCO usually occurs in postmenopausal or perimenopausal women, with ages ranging from 27 to 78 years old and a median age of 56 years. Clinically, HCO is associated with non-specific symptoms, including abdominal pain (53.2%), bloating (42.5%), pelvic mass (25.5%), weight loss (10.6%), and vaginal bleeding (8.5%). This case involves a 44-year-old woman who presented with persistent irregular vaginal bleeding as the initial symptom—an age notably younger than the median age of onset reported in the existing literature. The patient also had a long-standing history of chronic hepatitis B virus (HBV) infection. These findings highlight the importance of considering HCO in female patients presenting with abnormal vaginal bleeding, particularly in the context of chronic HBV infection and elevated serum AFP levels.

**Table 1:** Hepatoid carcinoma of the ovary literature review.

Author	Age	Symptoms	Site,size(cm)	Surgery	Treatment	Prognosis	FIGO stage
H Ishikura, R E Scully(1987) (1)	42	Pelvic peritonitis	L 6x5, R 5x4	TAH + BSO + AP	Chemoradiation	DOD, 5 years	IIB
H Ishikura, R E Scully(1987) (1)	71	Abdominal distension	L 20	TAH + BSO + AP+Om	Radiation	NED, 2 years	IIIC
H Ishikura, R E Scully(1987) (1)	57	Abdominal distension	R 10.5x7.5x5.5	TAH + BSO	ND	DOD, 4 months	IIIC
H Ishikura, R E Scully(1987) (1)	78	Abdominal distension and cramping	ND/ND	BSO + POm + colectomy	Melphalan (1 dose)	DOD, 4 months	IIIC
H Ishikura, R E Scully(1987) (1)	68	Abdominal pain,pelvic mass	R 10x6x5	BSO	Chemoradiation	DOD, 10 months	III
Matsuta, M;et al(1991) (5)	64	Abdominal mass	R 18x17x16	TAH + BSO + Om	IP cisplatin;chemotherapy	NED, 2 years	IA
Badreddine, J;et al(1993) (6)	52	ND	ND	ND	Carboplatin/cyclophosphamide/cisplatin	AWD, 7 months	III
Tamakoshi, K;et al(1993) (7)	62	Abdominal pain	R 8.2x7.8x6.4	TAH+BSO+BPL	Bleomycin/vincristine/cisplatin;cisplatin/etoposide;cyclophosphamide/mitomycin/5-fluorouracil	DOD, 13 months	IA
Nishida, T;et al(1995) (8)	43	Abdominal mass and pain	L 6x7x7, R 6x6x8	TAH + BSO + Om+RL	Cisplatin/epirubicin/ifosfamide	NED, 2 years	IIIC
Scurry, J P;et al(1996) (9)	72	Abdominal distension,dyspnea, lethargy	L 9.5, R 5.5	TAH + BSO + Om + right hemicolectomy	Carboplatin	AWD, 6 months	III
Trivedi, P;et al(1998) (10)	53	Abdominal mass and pain	L 9x8x6, R 8x7x6	TAH + BSO + Om	Cisplatin/cyclophosphamide	NED, 12 months	III
Maymon, E;et al(1998) (11)	35	Lower abdominal mass	L 35x30	L-SO + omental biopsy + AHT + R-SO + Om	Cyclophosphamide/cisplatin/carboplatin/etoposide;paclitaxel	DOD, 22 months	IIIA
Senzaki, H; et al(1999) (12)	61	Abdominal distension	L 12x9	TAH+L-SO+POm	IP cisplatin;chemotherapy(cisplatinum/5-fluorouracil/etoposide)	DOD, 20 months	III
Lee, Chao-Hsi;et al(2002) (13)	64	Abdominal pain	R 23x17x16	ATH+BSO+BPL+Om+small	Cisplatin/cyclophosphamide;cisplatin/paclitaxel/radiation;cisplatin/paclitaxel	DOD, 5 years	IIIC
Watanabe,Yoh;et al(2003) (14)	36	Abdominal pain	L 10x8x8	ATH+BSO+Om+BPL	Chemotherapy(bleomycin/etoposide/cisplatin)	ND	IIIC
Tochigi, Naobumi;et al(2003) (15)	69	Postmenopausal bleeding,abdominal mass	L 12	L-SO	ND	ND	IA
Tochigi, Naobumi;et al(2003) (15)	53	Ovarian mass	L 10	ND	Paclitaxel/Carboplatin	NED, 13 months	IIB
Tochigi, Naobumi;et al(2003) (15)	76	Ovarian mass	L 16	TAH+BSO+partialcolectomy	ND	NED, 4 years	IIB
Tsung, J S H;Yang, P S(2004) (16)	57	Abdominal pain	R 13x9x8	TAH + BSO + Om	ND	NED, 3 years	ND
Yiğit, S;et al(2006) (17)	63	Postmenopausal bleeding,abdominal pain	R 16x12	TAH + BSO +POm	Cisplatin/cyclophosphamide	NED, 7 months	IA
Kwon, J E;et al(2006) (18)	40	Abdominal distension,amenorrhea	R 11x9.5x3	ATH+SO+Om	Chemotherapy	NED, 6 months	III
Lazaro, Jesus;et al(2007) (19)	42	Abdominal pain	R 17x6	ATH+BSO+AP+Om+BPL	Carboplatin/paclitaxel	DOD, 16 months	IA
Ozan, H;et al(2008) (20)	50	Abdominal distension	L 10x8, R 7x6	ATH + BSO+Om+Tumor excision from the pelvis and ileostomy	Cisplatin/paclitaxel;cisplatin/gemcitabine;doxorubicin	DOD, 2 years	IIIC
Tejerina González, Eva;et al(2008) (21)	65	Abdominal distension,anorexia,and progressive dyspnea	R 12x10x6	ATH+BSO+Om	Cisplatin/paclitaxel	ND	III
Zizi-Sermpetzoglou, A;et al(2009) (22)	42	Abdominal pain	L 11x7x7	ATH+BSO+Om	ND	ND	ND
Isonishi, Seiji;et al(2009) (23)	59	Abdominal distension	L 18x15x16	ATH+BSO+ POm+low anterior resectionof rectum	Chemotherapy(bleomycin/etoposide/cisplatin)	ND	III
Sun J;et al(2009) (24)	34	Abdominal distension	R 14x10.5x8	ND	Chemotherapy	ND	IIA
D’Antonio, Antonio;et al(2010) (25)	42	Abdominal pain,pelvic mass	L 6x4x3	L-SO/ATH+R-SO+Om+AP	Chemoradiation	ND	I
Pandey, Manjari;et al(2011) (26)	46	Weight loss,abdominal pain,increasing abdominal girth	L 4.5, R 6.5	ATH+BSO+Om+tumor debulking	Chemotherapy(carboplatin/paclitaxel/sorafenib)	ND	III
Liu, Xin-Li;et al(2012) (27)	55	Abdominal pain,abdominal distention,increasing abdominal girth	L 11x8x7	ATH+BSO+Om+tumor debulking	Chemotherapy(docetaxel/nedaplatin)	NED, 10 months	IIIC
Sung, Ji-Hee;et al(2013) (28)	51	Abdominal pain,hematochezia	R 9x8x6	ATH+BSO+Om+AP+sigmoidectomy+ tumor debulking	Chemotherapy(paclitaxel/Carboplatin)	DOD, 6 months	IVB
Cascales Campos;et al(2013) (29)	57	Abdominal discomfort and distension,nauseas,vomiting, weight loss	ND 12x12x12	ATH+BSO+Om+AP+tumor debulking	IP paclitaxel;Carboplatin/paclitaxel;Radiation	NED, 28 months	IIIC
Wang, Lina;et al(2013) (30)	53	Abdominal distension	L 7x7x6, R 9x7x6	BSO+Om+tumordebulking	Chemotherapy(Carboplatin/paclitaxel)	NED, 15 months	IIIC
Wani, Nahida;et al(2013) (31)	58	Abdominal pain,abdominal distension,weight loss	L 4x2.4x1.6	ATH+BSO+Om+AP+tumor debulking	Neoadjuvant chemotherapy(Carboplatin/paclitaxel),chemotherapy	ND	ND
Mazouz, Aicha;et al(2015) (32)	78	Weight loss,umbilical mass	L 3.9x4.1, R 9.9	ND	Palliative Chemotherapy(Carboplatin/paclitaxel)	DOD, 1 months	IVB
Randolph, Laura K;et al(2015) (33)	73	Abdominal distension,weight loss,abdominal mass	L 24x16.5x13	ATH+BSO+small bowel resection	Chemotherapy(Carboplatin/paclitaxel)	NED, 26 months	IIIC
Lakhotia, Manoj;et al(2016) (34)	47	Abdominal pain,fatigability,lethargy,breathlessness	R 10x10x7	ATH+BSO	Chemotherapy	DOD, 3 months	ND
Naffouje, Samer A;et al(2016) (35)	47	Vague lower abdominal pain	ND	Leftpartial hepatectomy+tumor debulking	Hyperthermic intraperitoneal chemotherapy (HIPEC:cisplatin)	NED, 22 months	ND
Mahmood, Humera;et al(2017) (36)	41	Abdominal pain and distension,anorexia	L 3x5	Core biopsy of omental nodule	Sorafenib;progress:chemotherapy (paclitaxel/carboplatin)	NED, 2 months	IIIC
Ghosh, Joydeep;et al(2020) (37)	56	Abdominal distension, loss of appetite	ND	ATH+BSO+Om+BPL+PAoL+tumor debulking	Chemotherapy (carboplatin/paclitaxel;second-line therapies: doxorubicin;progress:sorafenib)	DOD, 9 months	IVB
Choi, Won-Ku;et al(2020) (3)	65	Abdominal distension and indigestion	L 13	tumor debulking	Chemotherapy(carboplatin/paclitaxel),radiation and palliative Chemotherapy(Carboplatin/gemcitabine)	DOD, 31 months	IC
Uribe Rivera, Ana Karla;et al(2020) (38)	27	Abdominal pain	R 10	Conservative surgical ovarian staging(SO+PC+BPL+PAoL+IOm+PB)	Chemotherapy (bleomycin/etoposide/platinum)	NED, 3 years	IA
Liu, Yao;et al(2021) (39)	66	Abdominal distension and pain	B omental mesentery, and peritoneal implants	ATH+BSO+Om+tumor debulking	Chemotherapy (carboplatin/docetaxel)	DOD, 3 months	IIIC
Liu, Yao;et al(2021) (39)	48	Abdominal mass and pain	L 9.5x8.8	ATH+BSO+AP+Om+BPL+PAoL	Chemotherapy(carboplatin/paclitaxel)	NED, 22 months	IC2
Li, Jiana;et al(2023) (2)	64	Abdominal pain	L 3.3x3x1.5, R 9x7x4	ATH+BSO+AP+Om+BPL+tumor debulking	Chemotherapy(carboplatin/paclitaxel)	DOD, 36 months	IIIC
Xiaofang Zhang;et al.(2024) (40)	67	Abdominal distension	Circular ligament of liver: 2, appendix mesangial root: 3	TAH+BSO+Om+AP+tumor debulking	Neoadjuvant therapy (paclitaxel/nedaplatin/bevacizumab); chemotherapy (paclitaxel/carboplatin;bevacizumab) Maintenance Therapy (niraparib/anlotinib)	NED, 30 months	IIIC
Present case(2025)	44	Irregular vaginal bleeding	R 9	ATH+BSO+Om+tumor debulking	Chemotherapy(paclitaxel/Carboplatin);targeted therapy (bevacizumab)	NED, 25 months	IIIC

Although HCO typically presents with unilateral ovarian involvement, bilateral disease is rare. In this case, intraoperative findings revealed bilateral ovarian lesions accompanied by peritoneal implantation metastases, with the disease staged as FIGO stage III. This aligns with previous reports indicating that HCO is frequently diagnosed at an advanced stage, with up to 55% being stage III. This presentation underscores the aggressive nature and early metastatic potential of HCO, emphasizing the need for heightened clinical awareness and prompt diagnostic evaluation in suspected cases.

Pathological diagnosis remains the gold standard for identifying HCO. Accurate diagnosis requires an integrative evaluation of patient age, clinical history, tumor marker levels, histopathological characteristics, and immunohistochemical profiles. HCO is characterized by tumor cells with abundant eosinophilic cytoplasm, a hallmark feature closely resembling HCC. Although AFP immunohistochemical positivity is a frequently utilized diagnostic marker, it is not universally present, as AFP-negative HCO cases have also been documented (26, 27). This underscores the necessity of a multidisciplinary diagnostic strategy, particularly when AFP levels are normal.

Due to the non-specific clinical presentation of HCO and the fact that elevated AFP levels can be observed in various ovarian and metastatic tumors, establishing a differential diagnosis can be highly challenging. Clinically, HCO must be distinguished from a range of common gynecologic conditions and malignancies. A comprehensive assessment that integrates the patient’s age, clinical symptoms, imaging findings, and laboratory test results is essential. In this case, the patient’s initial symptom was persistent irregular vaginal bleeding, a common symptom in perimenopausal women. Frequently associated conditions include endometrial carcinoma, endometrial hyperplasia, uterine fibroids, and dysfunctional uterine bleeding (41). In addition to uterine disorders, ovarian malignancies such as serous carcinoma and clear cell carcinoma may also present with symptoms like abdominal distension, pelvic masses, and abdominal pain, often due to ascites or tumor compression. Therefore, when middle-aged and elderly perimenopausal women present with the aforementioned symptoms, especially when routine gynecological examinations do not reveal obvious uterine abnormalities, the possibility of ovarian tumors should be highly suspected. In cases with elevated AFP levels, several differential diagnoses must be considered. First, yolk sac tumors (YSTs) typically occur in young women (usually before the age of 30) and commonly present with lower abdominal pain, palpable masses, and markedly elevated AFP levels. They are often associated with menstrual irregularities or primary amenorrhea, which differ from the persistent abnormal uterine bleeding seen in this 44-year-old perimenopausal patient. Histologically, YSTs often exhibit reticular or microcystic patterns and Schiller-Duval bodies, with strong immunopositivity for SALL4, Glypican-3+ and AFP, and negativity for HepPar-1. In this case, the tumor was negative for SALL4 and positive for HepPar-1, suggesting a non-germ cell origin and effectively ruling out YST (3, 4, 26, 39). Second, metastatic hepatocellular carcinoma (HCC) should be considered, particularly in the context of HBV infection, which may confound the diagnosis. HCC often presents with right upper quadrant pain, hepatic masses, abnormal liver function tests, and hepatic lesions on imaging. However, this patient had no hepatic symptoms or liver lesions, and the tumor was CK7-positive on immunohistochemistry, whereas HCC is typically CK7-negative. These findings favor a primary ovarian origin and exclude metastatic HCC (42, 43). Additionally, Krukenberg tumors—metastatic ovarian tumors originating from gastrointestinal malignancies—commonly present with gastrointestinal symptoms such as anorexia, nausea, diarrhea, or hematochezia, and are frequently associated with ascites. Imaging typically shows bilateral ovarian involvement. Although this patient also had bilateral ovarian lesions, there were no gastrointestinal symptoms, colonoscopy results were unremarkable, and immunohistochemistry was negative for CK20 and CDX2, ruling out gastrointestinal metastasis (29). Lastly, ovarian clear cell carcinoma (CCC), more common in postmenopausal women, usually presents as a pelvic mass with mild AFP elevation. Symptoms are mainly due to tumor compression or mass effect. Histologically, CCC is characterized by clear cytoplasm and vacuolated nuclei, with typical positivity for Napsin A and HNF-1β, and negative HepPar-1 staining. In contrast, the tumor in this case exhibited eosinophilic cytoplasm, partial AFP positivity, and HepPar-1 positivity, arguing against CCC (44, 45). In summary, considering the patient’s perimenopausal age, initial symptom of persistent irregular vaginal bleeding, significantly elevated AFP, bilateral ovarian lesions without hepatic involvement, and the tumor’s immunohistochemical profile (CK7+, HepPar-1+, Glypican-3+, SALL4–), the clinical and pathological findings support a diagnosis of HCO. Clinicians should be alert to the possibility of HCO in postmenopausal women presenting with abnormal vaginal bleeding and elevated AFP levels to avoid misdiagnosis as more common ovarian tumors or metastatic cancers.

Given the lack of specific clinical features, accurate and early diagnosis of HCO relies on a comprehensive approach integrating clinical evaluation, imaging studies, and pathological immunohistochemistry, which also facilitates the development of individualized treatment strategies.

Multidisciplinary discussions (MDTs) play a key role in guiding the diagnosis and treatment of HCO, especially in the absence of a therapeutic consensus for rare diseases. Currently, standardized treatment guidelines or a globally consistent framework for HCO have not been established. However, the majority of patients undergo cytoreduction followed by adjuvant chemotherapy, a strategy analogous to that used for epithelial ovarian cancer. Surgical interventions were performed in almost all reported cases (91.5%), including salpingo-oophorectomy (85%), total hysterectomy (74%), omentectomy (68%), appendectomy (21%), bilateral pelvic lymphadenectomy (17%), colectomy or hemicolectomy (9%), and para-aortic lymphadenectomy (6%). Forty-one of these patients (87%) received chemotherapy. The most common regimens include paclitaxel combined with platinum agents (45%) and cyclophosphamide combined with platinum agents (11%). Notably, given the hepatocellular differentiation observed in HCO, some studies have explored the use of first-line targeted therapies typically employed in hepatocellular carcinoma. Three patients (6%) were treated with sorafenib, an oral multi-targeted tyrosine kinase inhibitor approved for the treatment of advanced HCC (46). Additionally, two patients received targeted therapy with bevacizumab, a first-line agent commonly used for advanced HCC (47).

Of note, two patients who received bevacizumab in combination with paclitaxel-platinum-based chemotherapy demonstrated survival durations of 30 and 25 months (as described in the present case), indicating a potential survival benefit associated with the therapy. However, the limited clinical data available are insufficient to definitively support its efficacy. In contrast, none of the three patients treated with sorafenib exhibited a significant survival benefit, and one of them experienced disease progression within 2 months of treatment. This indicates that sorafenib may exhibit limited efficacy in this patient population, but further studies are needed to elucidate its applicability in specific clinical settings.

Genetic testing in this patient identified a TERT promoter mutation (variant allele frequency: 22.07%) and a PIK3C3 gene mutation (variant allele frequency: 25.81%). The TERT promoter mutation has been well-documented to correlate with increased aggressiveness and telomerase activation in HCC (48), suggesting that HCO may share molecular features with HCC. As a member of the class III phosphoinositide 3-kinase (PI3K) family, PIK3C3 plays a critical role in the regulation of autophagy (49), and its mutation may disrupt metabolic homeostasis in tumor cells, potentially offering a novel avenue for targeted therapy (50). Although the molecular landscape of HCO remains poorly characterized, the identification of these mutations highlights the potential of multi-omics approaches to elucidate key oncogenic drivers and uncover new targets for personalized treatment strategies.

Furthermore, the role of maintenance therapy in HCO remains uncertain due to the limited number of reported cases with BRCA mutations or homologous recombination deficiency (HRD). Notably, one patient achieved a progression-free survival (PFS) of 30 months following maintenance therapy with niraparib in combination with the multitargeted anti-angiogenic agent anlotinib, and remains under follow-up (40). This case suggests that a subset of HCO patients may be sensitive to PARP inhibition. These findings underscore the importance of comprehensive genetic testing in patients with HCO to identify potential therapeutic targets and to inform individualized treatment strategies.

Prognostic factors in HCO remain poorly defined due to the limited number of cases, but emerging patterns suggest that advanced FIGO stage, older age, bilateral ovarian involvement, and peritoneal or distant metastases are associated with worse outcomes. Early detection and aggressive cytoreductive surgery are crucial for improved prognosis. Given the high risk of recurrence, close surveillance—including periodic CT or PET/CT imaging and serial AFP monitoring—is essential. In the present case, although no radiologic recurrence was found during 25 months of follow-up, progressively rising AFP levels suggested possible biochemical relapse, highlighting AFP’s role as a dynamic biomarker.

Literature analysis indicates that HCO is associated with a poor overall prognosis, with approximately 36% of patients dying from disease progression. Peritoneal metastasis is the most common cause of death, followed by hepatic and bone metastases. Notably, a rare case of bone marrow metastasis has also been reported, resulting in fatality (34). Nineteen patients (40%) remained disease-free after treatment, while 2 patients (4%) experienced relapse at the last follow-up visit. Survival time ranged from 1 month to 5 years, with a median follow-up of 17 months. In a noteworthy pregnancy-associated case, a 35-year-old patient diagnosed at 23 weeks’ gestation delivered a baby boy by cesarean section at 33 weeks, with a 5-minute Apgar score of 9. Unfortunately, the patient passed away 22 months after the initial diagnosis due to pelvic and lower abdominal recurrence with liver metastases (11). This case emphasizes the necessity of a multidisciplinary approach to balance maternal and infant safety with effective tumor control.

This case is particularly notable for the younger age of onset, bilateral ovarian involvement, prolonged follow-up, molecular analysis, and positive response to a combination regimen including bevacizumab. These features provide meaningful insights into the potential molecular underpinnings and therapeutic responsiveness of HCO. Moving forward, systematic molecular analysis and long-term data collection from similar cases are warranted to define actionable targets and establish evidence-based treatment strategies for this rare malignancy.

## Conclusion

Hepatoid carcinoma of the ovary (HCO) is an exceptionally rare and aggressive malignancy that presents considerable diagnostic and therapeutic challenges. Consolidating clinical experiences from reported cases is critical to advancing our understanding of its clinical manifestations, histopathological features, and optimal treatment strategies. In this case, the diagnosis was confirmed through a multidisciplinary approach, and the patient responded favorably to a treatment regimen combining cytoreductive surgery, platinum-based chemotherapy, and bevacizumab, highlighting the potential applicability of this multimodal strategy in advanced HCO. Notably, molecular analysis identified a somatic PIK3C3 mutation, suggesting a possible link to tumor angiogenesis and providing a rationale for anti-angiogenic therapy. This case contributes valuable clinical and molecular insight that may inform future therapeutic decisions.

Looking ahead, the integration of molecular profiling into the diagnostic and therapeutic workflow for rare ovarian tumors such as HCO may facilitate the development of precision medicine approaches. Collaborative efforts to expand global case registries and genomic data repositories are urgently needed to establish evidence-based treatment guidelines and improve patient outcomes.

## Data Availability

The original contributions presented in the study are included in the article/**Supplementary Material**. Further inquiries can be directed to the corresponding author/s.

## Supplementary material

Supplementary Figure 1 Diagnostic process.

Supplementary Figure 2 Whole course of disease monitoring using AFP, CA125, and CA724 values.

Supplementary Figure 3 Timeline of the patient's entire treatment process.
