# Supplementary material for: Case Report: A rare case of hepatoid carcinoma of the ovary with genomic profiling and long-term follow-up: diagnostic and therapeutic perspectives
Source: Front Oncol. 2025 Aug 22;15:1631424. doi: 10.3389/fonc.2025.1631424 (PMC12412033; doi:10.3389/fonc.2025.1631424)

Supplement Figure 1. Diagnostic process


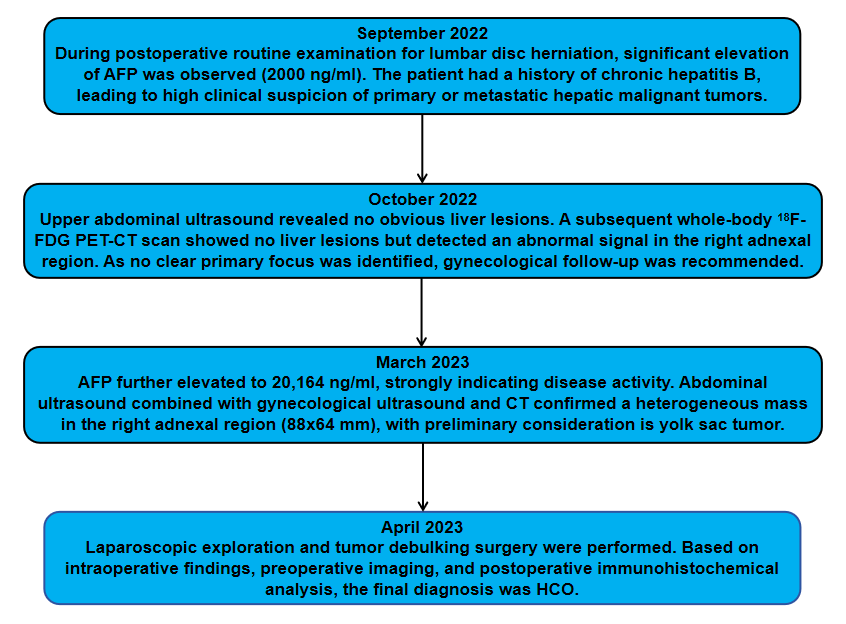


Supplementary Figure 2. Whole course of disease monitoring using AFP, CA125, and CA724 values.

A.


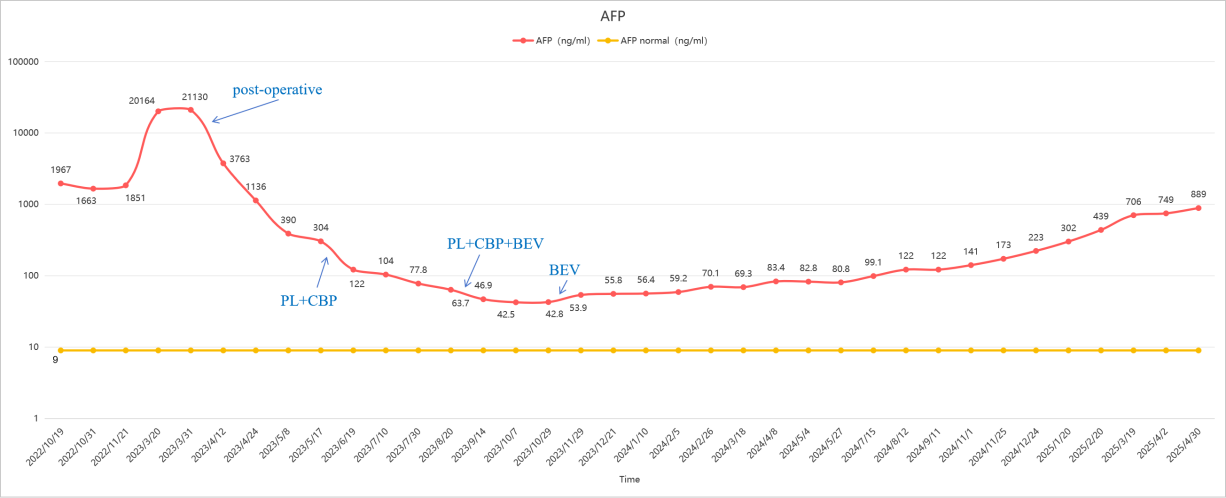


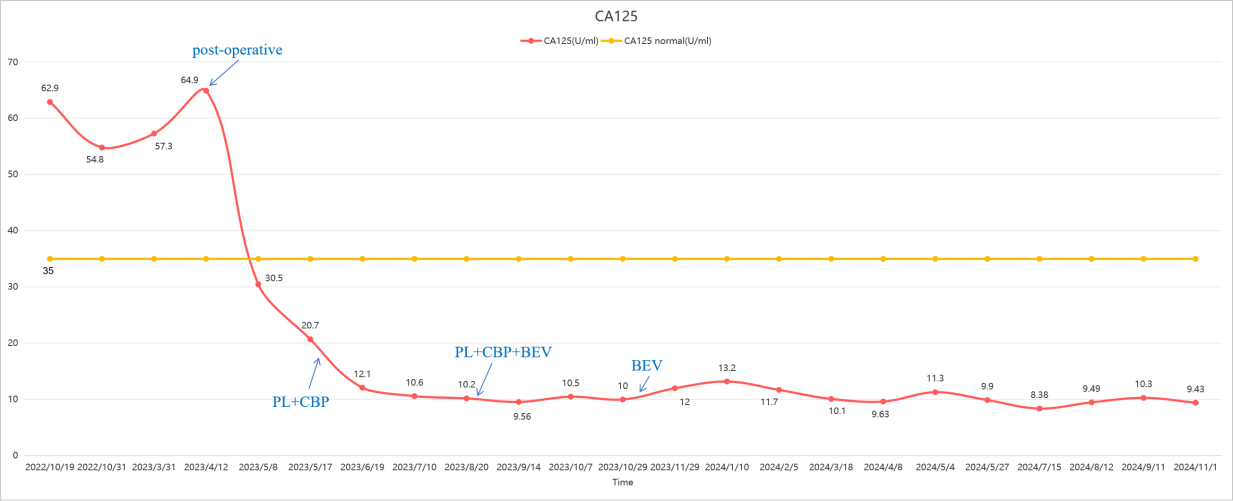


C.
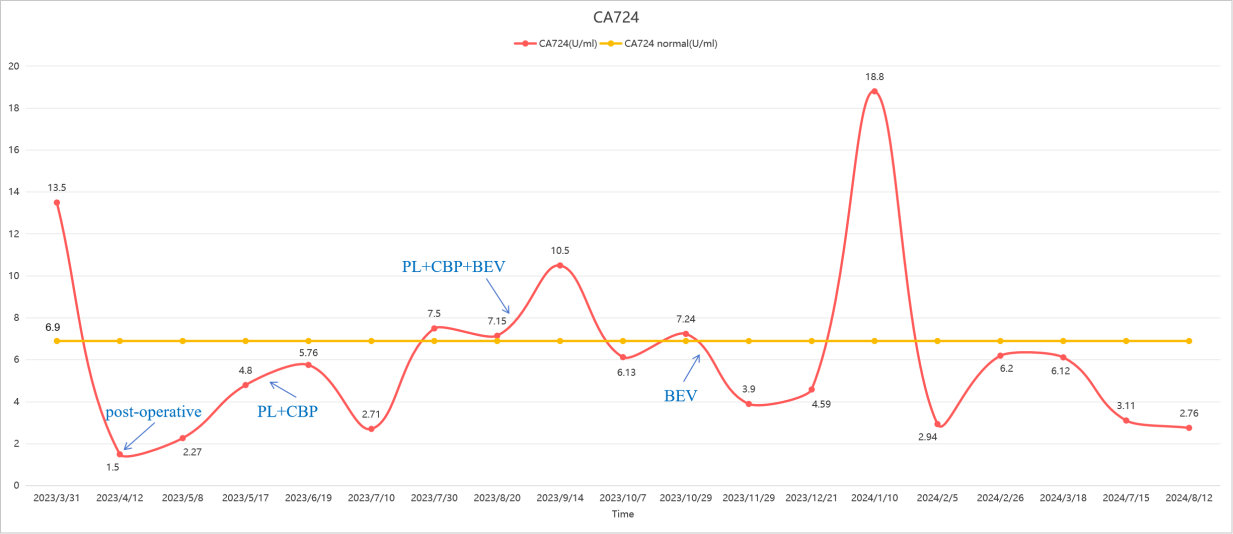


Supplementary Figure 3.Timeline of the patient's entire treatment process.


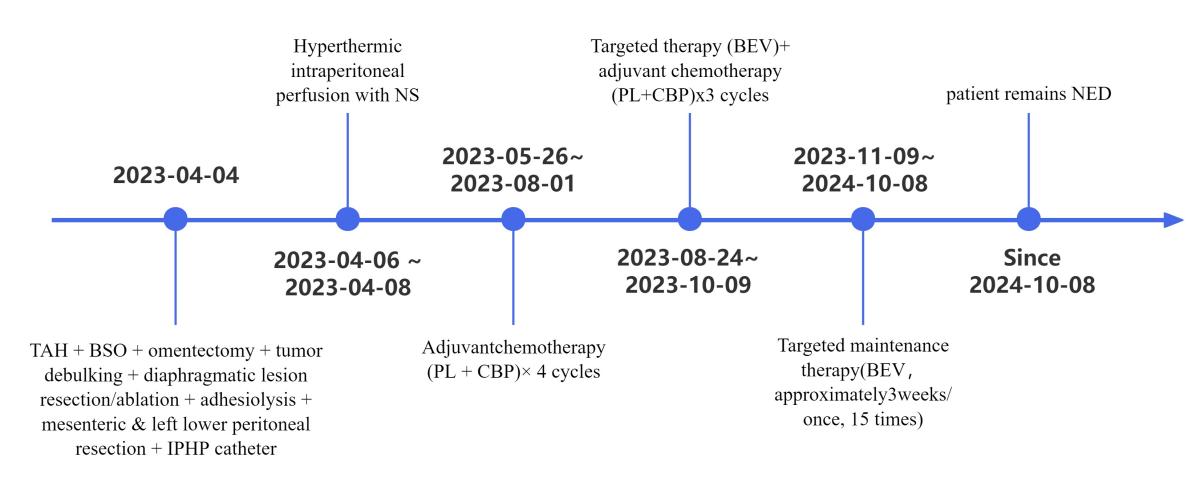

Supplement: Supplementary Figure 1 — Diagnostic process. [file DataSheet1.docx]
